# Validation of a Smart Ring Oximeter in Individuals With Dark Skin Pigment

**DOI:** 10.1016/j.mcpdig.2023.06.012

**Published:** 2023-07-28

**Authors:** Miguel Meira e Cruz, Congcong Zhou, Meir H. Kryger, Hongwei Wang

**Affiliations:** a Sleep Unit, Centro Cardiovascular da Universidade de Lisboa, Lisbon School of Medicine, Lisbon, Portugal; b European Sleep Center, Lisbon, Portugal; c School of Medicine, Sir Run Run Shaw Hospital, Zhejiang University, Zhejiang, China; d Zheijang University, Biomedical Sensors National Spacial Laboratory of Biomedical Engineering and Instrument Science, Zheijang, China; e Yale School of Medicine, New Haven, CT; f Tongde Hospital of Zhejiang province, Hangzhou, China

## Abstract

**Objective:**

To evaluate a ring wearable’s accuracy in the measurement of oxyhemoglobin saturation measured by pulse oximeter (SpO_2_) compared to a simultaneous SaO_2_ obtained from arterial blood gases (ABGs) because oximeters may overestimate oxyhemoglobin saturation in darkly pigmented individuals resulting in occult hypoxemia.

**Patients and Methods:**

Circul+ (Bodimetrics), with a form factor of a ring, measures several variables for cardiorespiratory assessment (SpO_2_, movement, and heart rate). The design ensures that measurements are made on the palmar surface of the digit, where there is generally less pigment. Twenty-four healthy participants (8 Black, 16 non-Black) had catheters inserted into their radial arteries during the timeframe between August 2020 and January 2022. They were administered nitrogen-rich air that made them hypoxic in steps to an SaO_2_ of 70%. ABGs was sampled at various levels of hypoxia, and simultaneous readings were obtained from the Circul+. We compared SpO_2_ from Circul with measurement of ABGs by a medical grade blood gas analyzer.

**Results:**

There was excellent correlation between SaO_2_ measured by the ring oximeter and ABGs in both Black (*y* = 1.0174*x* − 1.573; *R*^2^ = 0.9414) and Non-Black (*y* = 1.0209*x* − 2.5607; *R*^2^ = 0.9207) participants. No differences were found in comparing the intercept and slopes of the regressions. At ABG of 70% and 100%, the SaO_2_ measured by the ring was calculated to be 69.6% and 100.2% for the Black participants and 68.9% and 99.5% for the non-Black participants. Bland-Altman analysis found a bias (deviation from the mean) of 0.0 for the Black participants and −0.7% for the non-Black participants.

**Conclusion:**

Results from this study confirmed that Circul+ oximetry accuracy seems to be independent of skin tone, perhaps because measurements are made on the palmar surface of the digit, where there is generally less pigment.

Accuracy is critical for screening, diagnosis, and follow-up in medicine. In recent years, the development of wearable technology has been providing inexpensive tools for data collection and analysis. These tools, when validated, have potential utility for both research and clinical medicine.

Wearable devices (wrist worn, fingertip, and ring) have been developed that have the ability to continuously monitor relevant respiratory variables such as oxyhemoglobin saturation (SaO_2_). The major class of currently available pulse oximetry devices compute arterial oxyhemoglobin saturation from the ratio of the pulsatile to the total transmitted red light divided by the same ratio for infrared light transilluminating a finger.

Derived oxygen saturation (SpO_2_) should not be impacted by skin pigmentation. Although several studies reported no significant pigment-related interferences in portable pulse oximeters,^1^, ^2^, ^3^ some early authors published less accurate results in dark-skinned individuals,^4^ and now, many articles have indicated that there is a great reason for concern.^5^ The proliferation and widespread use of pulse oximeters by the public during the COVID pandemic and the recent articles highlighting unintended racial bias in the devices have raised alarms.^6^, ^7^, ^8^, ^9^, ^10^, ^11^, ^12^, ^13^, ^14^ In particular, the inaccuracies (eg, occult hypoxemia^12^) can negatively impact patient care.^7^ Occult hypoxemia has been defined as arterial blood oxygen saturation (SaO_2_) of less than 88% despite a pulse oximetry (SpO_2_) measurement of 92% or higher.^12^ False high oximeter measurements can negatively impact patient care.^15^ There are many oximeters currently marketed to both the public and the medical profession. Clinicians managing patients should have access to the validation data of the devices. Here we present the validation data of the Bodimetrics Circul+ oximeter.

We hypothesize that measuring on the palmar surface of digits, where there is generally less pigment, will result in acceptable SaO_2_ values without occult hypoxemia in people with dark skin tones.

## Patients and Methods

**Figure 1:**
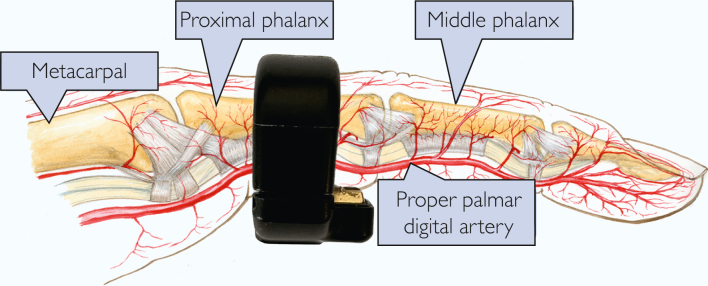
The sensors of the ring are close to the pulsating proper palmar digital artery.

The Circul+ (Bodimetrics) is a device with a form factor of a ring, which continuously measures several variables (SpO_2_ and heart rate by beat-by-beat reflectance photoplethysmography, and movement by accelerometer). The data are sampled beat by beat and processed into a 10-second moving window. The arterial blood samples and the corresponding SpO_2_ measurements were obtained using the following protocol. The ring has a patented design that assures that measurements are made on the palmar aspect of the finger (Figure 1). This work was performed at Tongde Hospital of Zhejiang province, China.

### Selection of Participants and Demographic characteristics

Data were obtained for as part of submissions of the Circul+ to the China Food and Drug Administration, currently the National Medical Products Administration. Volunteers were recruited by advertising and were students or hospital staff. Data were obtained from 24 participants (12 men and 12 women; 8 Black and 16 non-Black participants). The mean age was 22.58±2.71 years for men and 26±6.24 years for women. The Black patients were Fitzpatrick scale type VI.^16^ The others were of Han Chinese, mainly types I to IV. The research was approved by the ethics committee of Tongde Hospital, and all participants signed informed consent forms.

### Protocol and Data Acquisition

In a hospital setting, after topical anesthesia, participants had a catheter placed in a radial artery. Testing environment conditions were as follows: temperature 22-25 °C and humidity 50%-65%. The sensors (Circul+; Bodimetrics) for SpO_2_ and IntelliVue MX500/MX550 (Philips) for pulse rate and SpO_2_ were placed on fingers the same side as the arterial catheter. Each participant while breathing naturally was connected to an anesthesia breathing circuit with the inspired oxygen concentration adjusted to 21%. Tidal volume and breathing rate were monitored continuously. The relevant physiologic parameters, described below, were recorded. When the SpO_2_ was stable for at least 30 seconds and between 97% and 100%, 5 samples of blood (1-2 mL) were withdrawn with intervals of at least 20 seconds. The SaO_2_ of the samples was obtained by blood gas analysis (ABL90 FLEX blood gas analyzer; Radiometer), and the vital signs of the participant were observed and recorded. The participants’ inhaled oxygen concentration was then reduced by the progressive addition of nitrogen into the circuit. When the SpO_2_ reached 92%-96% and was stable for at least 30 seconds, 5 samples of blood (1-2 mL) were withdrawn during intervals of at least 20 seconds. As mentioned earlier, the SaO_2_ of the samples was obtained by blood gas analysis, and the vital signs of the participant were observed and recorded. As above, inhaled oxygen concentration was then further reduced sequentially so that measurements of 5 samples were made at each of the following ranges: 85%-91%; 78%-84%; and 70%-77%. Once all the data and samples had been collected, the participant-anesthesia machine interface was removed, and the participant breathed room air. After observing that the participant’s vital signs were normal, the arterial catheter was removed and the site pressurized to stop any bleeding, and the participant left the bed. After confirming that the participant was stable and physiological variables normal, at least 1 hour after leaving bed, the participant was discharged.

### Statistical Analyses

Statistical analysis was conducted using IBM SPSS (version 25.0, SPSS).

## Results

**Figure 2:**
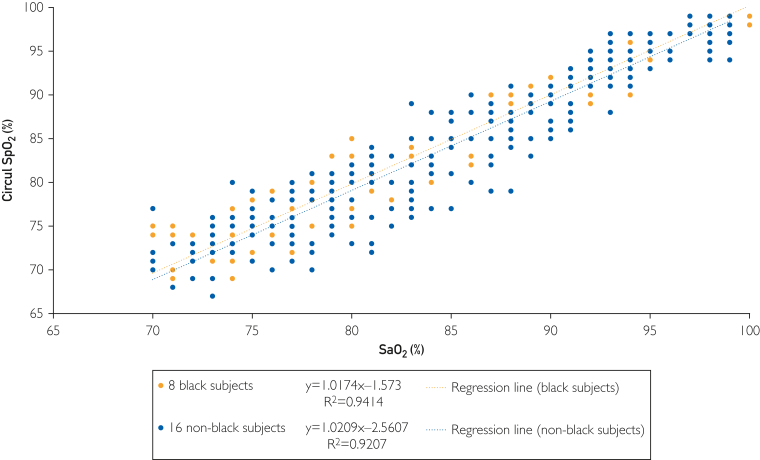
SpO_2_ measured by ring oximeter vs SaO_2_ measured by ABG. Hypoxemia was induced by lowering inhaled oxygen concentration.

**Figure 3:**
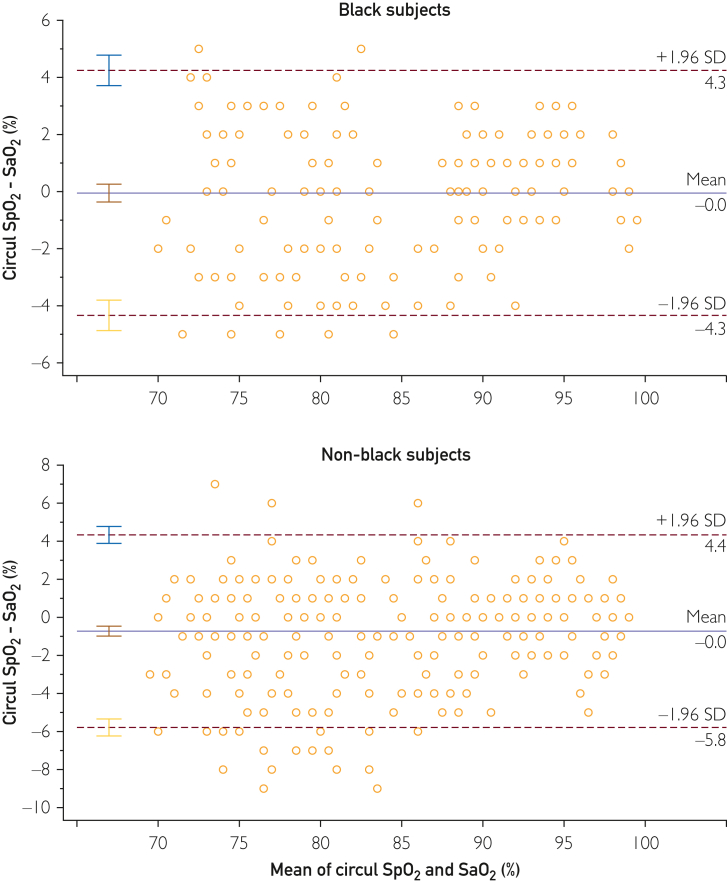
Bland-Altman plots for the Black (top) and non-Black (bottom) participants.

**Figure 4:**
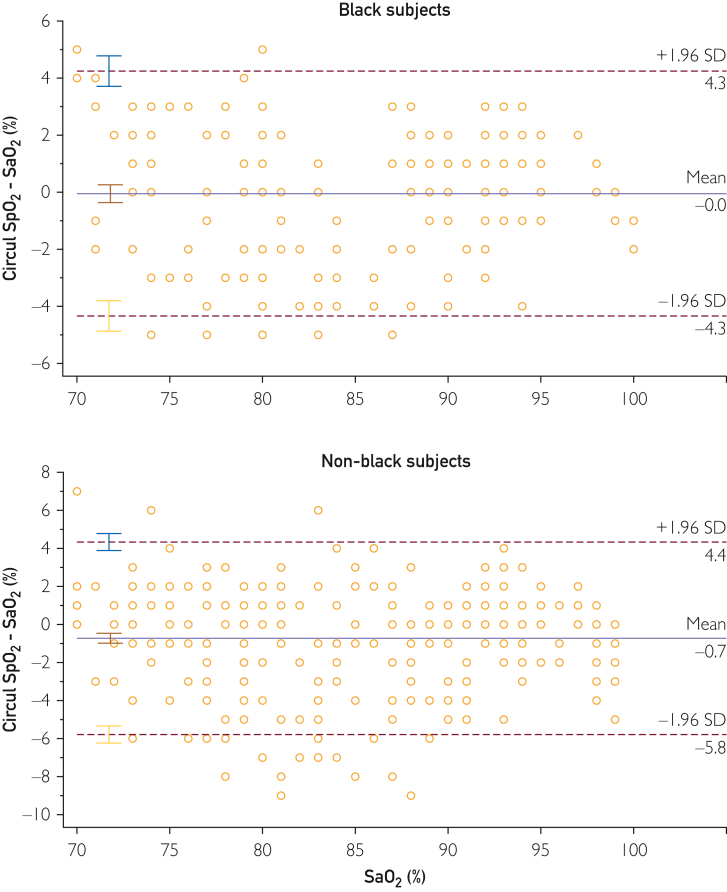
Modified Bland-Altman plots for the Black (top) and non-Black (bottom) participants.

**Figure 5:**
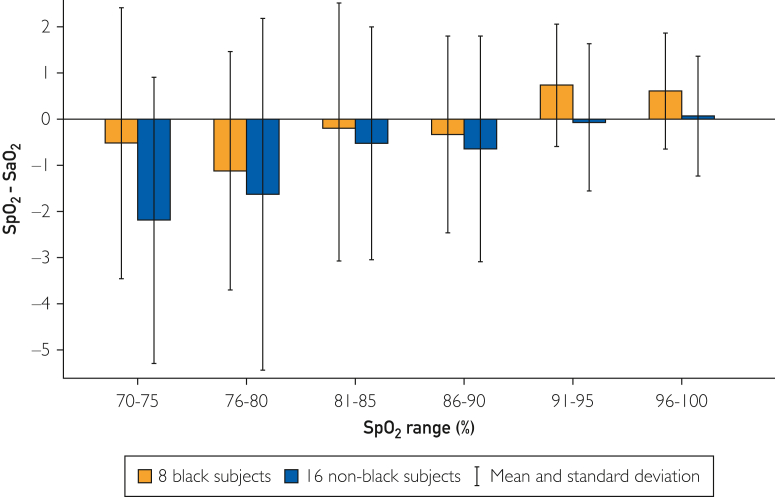
Box plot of (SpO_2_ − SaO_2_) by SpO_2_ range increments for all participants. Pulse oximeter readings were slightly lower than SaO_2_ when SaO_2_ was <80% for both Black and non-Black participants.

The data comparing Circul+ SpO_2_ and arterial blood gas (ABG)-obtained SaO_2_ in the Black and non-Black participants are reported in Figure 2. There was excellent correlation between SaO_2_ measured by the ring oximeter and ABGs in both Black (*y* = 1.0174*x* − 1.573; *R*^2^ = 0.9414) and non-Black (*y* = 1.0209*x* − 2.5607; *R*^2^ = 0.9207) participants. No relevant differences were found in comparing the intercept and slopes of the regressions. At ABG SaO_2_ of 70% and 100%, the SpO_2_ measured by the ring was calculated to be 69.6% and 100.2% for the Black participants and 68.9% and 99.5% for the non-Black participants, respectively. Figure 3 shows the Bland-Altman analyses of the Black and non-Black participants and Figure 4 shows analysis using a modified Bland-Altman technique. This latter analysis was done because the SaO_2_ measured by ABGs could be considered the gold standard. The Table summarizes the average root mean square error for the decadal ranges of SaO_2_. Figure 4 shows a box plot of (SpO_2_ − SaO_2_) by SpO_2_ range increments for all participants. This shows that the pulse oximeter readings were slightly lower than SaO_2_ when SaO2 was less than 80% for both Black and non-Black participants. Thus, severe hypoxemia would not be missed. This is corroborated in Figure 5, which shows the count of SaO_2_/SpO_2_ pairs by absolute value of the bias 3% or less, or more than 3% over SaO_2_ range. There were very few measurements in which SpO_2_ deviated by more than 3%.

**Table:** Average Root Mean Square Error

	SaO_2_ range		
	90%-100%	80%-89%	70%-79%
Non-Black participants	1.57	2.66	2.71
Black participants	1.73	3.84	2.84

Values are root mean square errors for the decadal ranges of SaO_2_

SaO_2_, oxyhemoglobin saturation.

**Figure 6:**
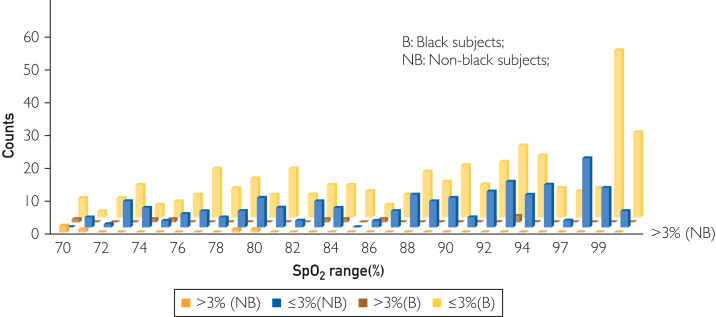
Count of SaO_2_/SpO_2_ pairs by absolute value of the bias ≤3% or >3% over the SaO_2_ range. There were very few measurements in which SpO_2_ deviated by >3% from measured SaO_2_ for both Black (green bars) and non-Black (blue bars) participants.

**Figure 7:**
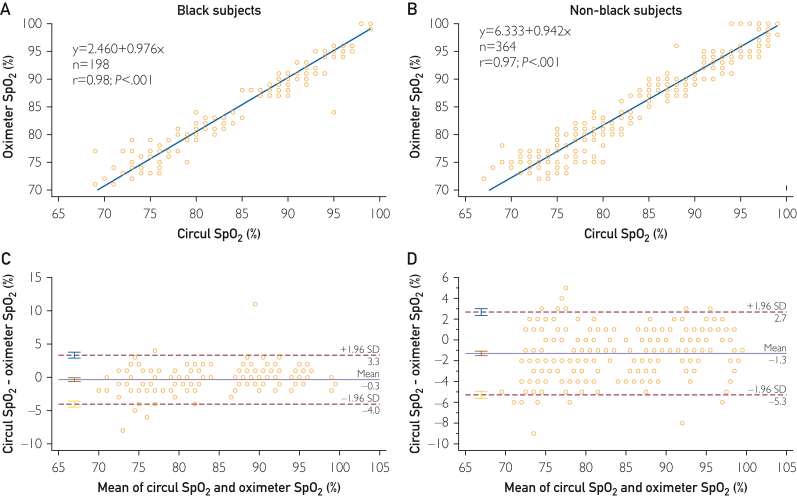
Comparison of the Circul oximeter with a medical grade oximeter using regression analyses (A and B) for Black and non-Black participants and Bland-Altman plots (C and D).

The data comparing the Circul with a medical grade finger oximeter are reported for SpO_2_ and pulse in Figures 6 and 7, respectively.

## Discussion

The Circul+ ring oximeter was found to be reasonably accurate in the measurement of SpO_2_ and pulse rate in both Black and non-Black participants in the range between 100% and 70% SaO_2_ when compared with ABGs (the gold standard) and a medical grade finger oximeter. Of great interest, there was not a single instance of occult hypoxemia (SpO_2_ > 92% when SaO_2_ was <88%). The explanation for its accuracy in Black participants may be related to the patented design that ensures that the readings are obtained from the palmar aspect of the finger, which generally has less pigment than the back of the hand. This device has been reported to be useful in screening for sleep apnea.^17^

There are many consumer oximeters on the market, and their validation data are generally not available.^18^ Most such oximeters available in the United States have not been cleared or approved by the US FDA. Food and Drug Administration approval or clearance (based on similarity to a predicate approved device) requires “10 or more healthy participants” and “participants with a range of skin pigmentations, including at least 2 darkly pigmented participants.”^19^ The inadequacy of this requirement is obvious, especially given the decades of knowledge about potential problems with oximeters.^4^^,^^20^^,^^21^ The first widely used oximeter, which was not a pulse oximeter (Hewlett Packard 407201A) was validated in a large number of Black patients and was found to accurate in this population.^22^^,^^23^

During the COVID pandemic, patients were often instructed to self-monitor with consumer oximeters at home rather than coming to hospitals.^24^ It appears that there was little thought given to potential pitfalls (eg, were the recommended devices accurate?) and at times inexplicable readings.^25^ The alarms raised first by Sjoding et al^10^ during the start of the COVID pandemic and more recent studies^7^^,^^9^^,^^11^^,^^12^^,^^15^ suggest that pulse oximeters may miss occult hypoxemia in patients with dark skin pigment. That unintended racial bias in oximetry, which was noted by the media, was an issue that led the Federation of American Scientists to convene a forum to review this issue.^26^

### Why Is the Circul+ Ring Less Affected by Dark Pigment?

**Figure 8:**
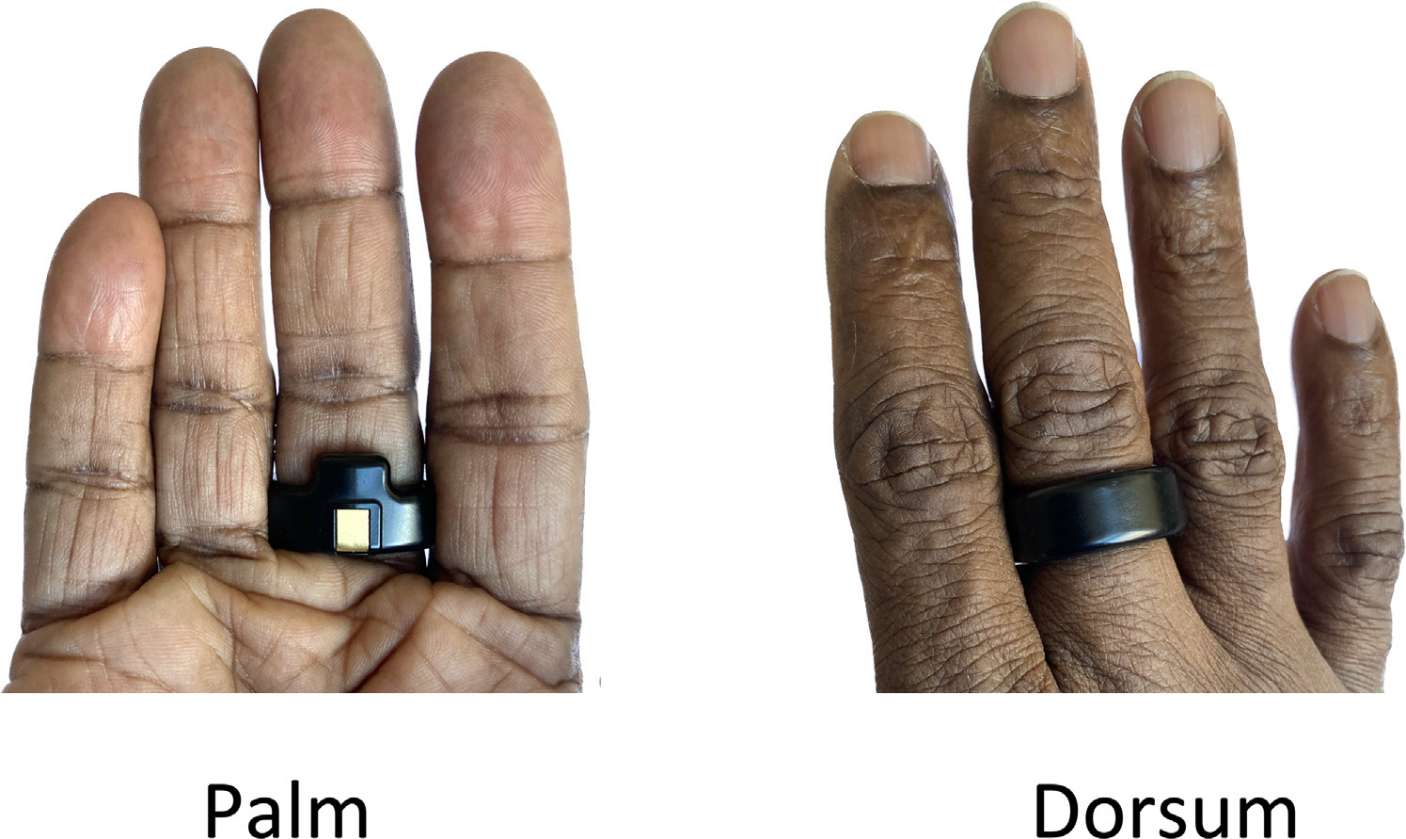
The palmar aspect of the hand is less pigmented than the dorsum. There is often increased pigment on the palmar aspect in the area of interphalangeal joints.

There are at least 2 possible explanations. First, as mentioned in Methods and shown in Figure 1, the sensors on the ring are on the palmar aspect of the finger closer to the palmar digital artery. Second, the palmar aspect of the hand usually has less pigment than the dorsum (Figure 8).

Oxygen saturation is a critical variable in management of patients with suspected or known hypoxemia. We believe that the validation data of all devices that measure SpO_2_ should be available to the prescriber and the user and that the pitfalls in measurement be understood by both. Even when validation is available, it is important to consider that validation studies are done in young healthy participants in controlled laboratory conditions. Clinicians should be aware of other well-known sources of SpO_2_ measurement errors and artifacts including those related to motion, medical conditions that might cause venous pulsations in tissue beds, poor peripheral perfusion that might occur in heart failure or shock, sensor placement and device sampling rate and signal filtering,^27^ nail polish, and abnormal hemoglobin (carboxyhemoglobin and methemoglobin) levels.^18^ SpO_2_ often overestimates SaO_2_ when blood pressure and heart rate decrease.^28^ Thus, the measurement of SaO_2_ with an ABG may be appropriate in some clinical situations.^28^

Technology is moving very quickly, and the boundaries between consumer and medical grade devices sometimes overlap. Wearables are being developed for monitoring in several medical fields.^29^, ^30^, ^31^, ^32^, ^33^ It is important that developers ensure that their products do not introduce unintended bias into the devices. It is also important that validation data be available.

## Conclusion

Results from this validation study confirmed that Circul+ oximetry accuracy seems to be independent of skin tone. Measurement in the palmar aspect of the digits (where there less pigmentation) may improve the accuracy of such devices in participants with dark skin tones.

## Potential Competing Interests

Dr Meira e Cruz is a scientific adviser for Bodimetrics. Dr Kryger is a scientific adviser for Bodimetrics, Dormothech, and Wesper. All other authors report no competing interests.
